# Microbial Mineralization with *Lysinibacillus sphaericus* for Selective Lithium Nanoparticle Extraction

**DOI:** 10.1021/acs.est.4c06540

**Published:** 2024-09-12

**Authors:** Toriana N. Vigil, Grayson C. Johnson, Sarah G. Jacob, Leah C. Spangler, Bryan W. Berger

**Affiliations:** † Department of Chemical Engineering, University of Virginia, Charlottesville, Virginia 22903, United States; ‡ Department of Chemical and Life Science Engineering, Virginia Commonwealth University, Richmond, Virginia 23284, United States

**Keywords:** biomineralization, mineral extraction, critical minerals, nanoparticles

## Abstract

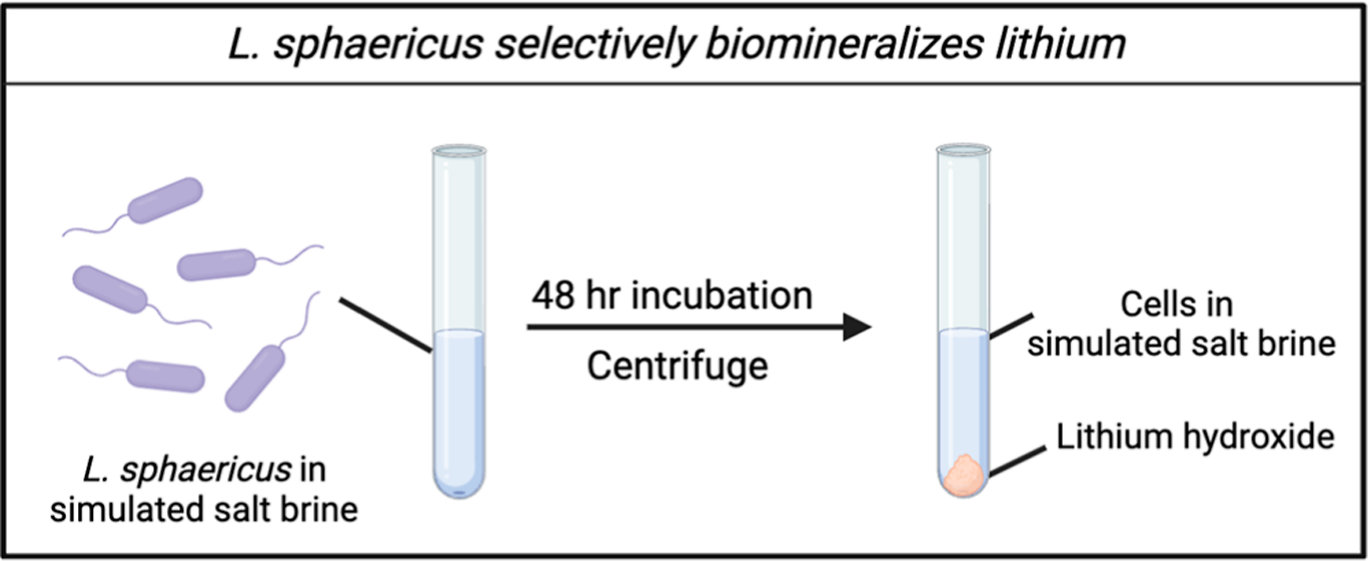

Lithium is a critical mineral in a wide range of current technologies, and demand continues to grow with the transition to a green economy. Current lithium mining and extraction practices are often highly ecologically damaging, in part due to the large amount of water and energy they consume. Biomineralization is a natural process that transforms inorganic precursors to minerals. Microbial biomineralization has potential as an ecofriendly alternative to current lithium extraction techniques. This work demonstrates *Lysinibacillus sphaericus* biomineralization of lithium chloride to lithium hydroxide. Quantitative analysis of biomineralized lithium via the 2-(2-hydroxyphenyl)-benzoxazole fluorescence assay reveals significantly greater recovery with *L. sphaericus* than without. Furthermore, *L. sphaericus* biomineralization is specific to lithium over sodium. The nanoparticles produced were further characterized via Fourier transform infrared and transmission electron microscopy analysis as crystalline lithium hydroxide, which is an advanced functional material. Finally, ESI–LC/MS was used to identify several proteins involved in this microbial biomineralization process, including the S-layer protein. Through the isolation of *L. sphaericus* ghosts, this work shows that the S-layer protein alone plays a critical role in the biomineralization of crystalline lithium hydroxide nanoparticles. Through this study of microbial biomineralization of lithium with *L. sphaericus*, there is potential to develop innovative and environmentally friendly extraction techniques.

## Abstract 2

Microbial biomineralization is used for sustainable critical mineral extraction to recover lithium salts as lithium hydroxide nanoparticles.

## Introduction

With the push for electrification comes an increasing need for industrially useful minerals, such as lithium, nickel, cobalt, and rare earth elements.^1^ The International Energy Agency predicts a 40× increase in lithium demand by 2050.^2^ While lithium deposits can be found worldwide, it is often economically and resource intensive to mine them,^3−5^ resulting in negative environmental and community impacts.^6−8^ Additionally, increased demand for lithium contributes to economic dependence and stress of the global supply chain.^9^ An alternative method to mining for lithium is the evaporative salt brine approach: targeting dissolved minerals found in natural bodies of water. With consideration to the high solubility of lithium, significant amounts of lithium can be found in bodies of water in contact with lithium-containing rocks.^1,3^ Due to their high salt content, these are often termed evaporative salt brines.^1,3^

Lithium can be collected as a slurry from evaporative salt brines as the liquid evaporates, and total dissolved solids become more concentrated. On average, to harvest 1 ton of lithium carbonate, kiloliters of concentrated brine (150 mg/L) are required.^3,4^ In fact, for brines that have relatively low concentrations of dissolved lithium species (<10 mg/L) extraction is even less efficient.^3,4^ After evaporation and concentration, the slurry is taken to specialized separation facilities for further refinement.^3^ While this is a potentially valuable source of lithium, this process consumes huge amounts of water, negatively impacting local environments as well as human and animal populations.^10^ Many direct lithium extraction techniques are being investigated with chemical precipitation, electrochemical ion pumping, and membrane technologies as a focus.^11−14^ While these direct lithium extraction techniques strive to be “greener” than current mining and evaporative brine approaches, only 30% of recent studies have targeted or used samples from existing lithium brines.^3^

Another promising approach for mineral extraction is biomineralization. Biomineralization is a natural process that converts inorganic precursors to a more stable inorganic mineral.^15^ Biomineralization sometimes occurs in microbes as an evolutionary adaptation to toxic levels of metals in their local environment.^16^ Microbially mediated biomineralization is specific, selective, and frequently produces physically complex structures.^16,17^ Furthermore, biological processes tend to generate less of an environmental impact than their chemical counterparts and can sometimes be used to remediate long-lasting chemical pollutants.^18^ To meet the world’s increasing demands for lithium, biomineralization is a highly promising green alternative to current extraction techniques. In this work, we have identified *Lysinibacillus sphaericus* as a promising microbe for lithium nanoparticle extraction and production of lithium hydroxide nanoparticles.

## Materials and Methods

*L. sphaericus* was isolated from a coal mine in Wise County, Virginia, United States of America. 16s rDNA sequencing (Azenta) showed that this *L. sphaericus* corresponds to DSM 28 (=ATCC 14577 = NBRC 15095 = KCTC 3346 = IAM 13420).

### Biomineralization with *L. sphaericus*

A saturated overnight culture of *L. sphaericus* in LB was centrifuged at 3000*g* for 10 min, and the supernatant was discarded. The cell pellet was resuspended in M9 minimal media [2 mM magnesium sulfate (Alfa Aesar), 0.1 mM calcium chloride (Alfa Aesar), 48 mM sodium phosphate dibasic (VWR), 22 mM monopotassium phosphate (J.T. Baker), 9 mM sodium chloride (VWR), 20 mM ammonium chloride (Sigma Life Science)], supplemented with a 170 mM final concentration lithium chloride (ICN Biomedicals Inc.), or sodium chloride (VWR) was added to simulate the co-occurrence of lithium and sodium species in a salt brine. Mixture was then incubated at 37 °C shaking for 48 h.

#### Lithium Quantification Assay

A 5 mL culture with *L. sphaericus* and 170 mM final concentration lithium chloride was incubated at 37 °C shaking for 48 h. Culture was transferred to a 50 mL centrifuge tube and spun at 10,000*g* for 30 min. The supernatant was discarded, and the pellet was dried. Pellet was resuspended in 200 μL of acetonitrile (Fisher Chemical). A stock solution of 1 mM triethylamine (Sigma-Aldrich) and 4 mM 2-(2-hydroxyphenyl)-benzoxazole (Tokyo Chemical Industry) in acetonitrile was used to make a standard curve with lithium chloride content of 0.1 μmol to 0 mol, and fluorescence was read at 392 nm excitation and 428 nm emission. Simple linear regression was used to determine line of best fit and the associated equation. 5 μL of the sample was combined with 145 μL of stock solution, and fluorescence was read at 392 nm excitation and 428 nm emission. The equation from the standard curve was used to calculate moles of lithium present in samples, which was then transformed to a fold increase as compared to the lithium only condition.

#### Sodium Quantification Assay

A 5 mL culture with *L. sphaericus* and 170 mM final concentration sodium chloride was incubated at 37 °C shaking for 48 h. Culture was transferred to a 50 mL centrifuge tube and spun at 10,000*g* for 30 min. The supernatant was discarded, and the pellet was dried. Pellet was resuspended in 200 μL of acetonitrile. A standard curve with sodium chloride content of 0.1 μmol to 0 mol was prepared in DI H_2_O and CoroNa Green (ThermoFisher) was added to all wells at a final concentration of 0.013 μM. After 30 min incubation at room temperature, fluorescence was read at 492 nm excitation and 516 nm emission. Simple linear regression was used to determine line of best fit and the associated equation. 5 μL of the sample was combined with 145 μL of stock solution, and CoroNa Green (ThermoFisher) was added to all wells at a final concentration of 0.013 μM. After 30 min of incubation at room temperature, fluorescence was read at 492 nm excitation and 516 nm emission. The equation from the standard curve was used to calculate moles of sodium present in samples, which was then transformed to fold increase as compared to the sodium only condition.

### Fourier Transform Infrared Spectroscopy

As with quantification assays, following *L. sphaericus* biomineralization with lithium chloride and/or sodium chloride, culture was transferred to a 50 mL centrifuge tube and spun at 10,000*g* for 30 min. The supernatant was discarded, and the pellet was dried. The pellet was resuspended in 200 μL of acetonitrile. A PerkinElmer FT-IR spectrometer “Frontier” with Universal attenuated total reflection Sampling Accessory (PerkinElmer) was blanked with acetonitrile followed by reading with the sample.

#### Transmission Electron Microscopy

Mineralized lithium was precipitated via centrifugation at 20,000*g* for 30 min. The supernatant was discarded, and precipitates were dried and then resuspended in 10 mM citrate buffer (pH 3.0). 100 μL of samples was applied to TEM sample grid and let dry at room temperature. Samples were examined with an FEI Titan 80-300 transmission electron microscope. Image analysis and particle size measurements were conducted with an ImageJ.

### Sodium Dodecyl Sulfate-Polyacrylamide Gel Electrophoresis

A 50 mL culture with *L. sphaericus* and 170 mM final concentration lithium chloride was incubated at 37 °C shaking for 48 h. Culture was transferred to a 50 mL centrifuge tube and spun at 10,000*g* for 30 min. The supernatant was discarded, and the pellet was dried. The pellet was gently resuspended in acetonitrile, diluted to 20% with DI H_2_O then lyophilized for approximately 48 h. The lyophilization product was a yellow powder. The lyophilization product was resuspended in 20 mL of DI H_2_O, then approximately 20 μL was loaded on a standard 8% resolving SDS PAGE gel. The gel was set at 110 V for 10 min and then 150 V for 1 h. The gel was removed and stored in a sealed bag with DI H_2_O at 4 °C and then sent for analysis via ESI–LC-MS/MS.

### Ghost Isolation

Ghost separation protocol was adapted from Pfeifer et al. (2022).^19^ One *L. sphaericus* colony was selected for a starter culture in LB, then grown to saturation overnight at 37 °C shaking. Cells were pelleted via centrifugation at 2000*g* for 30 min. The supernatant was discarded, and the cell pellet was resuspended in 1/5 original culture volume with buffer A [10 mM sodium chloride, 0.5% w/v sodium lauroylsarcosine (Fisher Scientific)] and then incubated at 37 °C shaking for 1 h. Then, phenylmethylsulfonlyfluoride (Sigma) and DNase I (Millipore Sigma) were added for final concentrations of 1 mM and 10 μg/mL, respectively. Solution was centrifuged at 20,000*g* for 30 min. The supernatant was discarded, and the pellet was resuspended in equal volume buffer B [10 mM sodium chloride, 0.5 mM magnesium sulfate, and 0.5% w/v sodium dodecyl sulfate (VWR)] followed by incubation at 40 °C shaking for 1 h. Solution was centrifuged at 20,000*g* for 30 min. The supernatant was discarded, and the pellet was resuspended in equal volume buffer B. Incubation and centrifugation steps with buffer B were repeated total of three times. After final centrifugation and discarded supernatant, the pellet was rinsed gently with DI H_2_O and then resuspended. Concentration was determined via A280 with extinction coefficient 177,600 M^–1^ cm^–1^.^19^ To confirm ghost isolation, the sample was examined via sodium dodecyl sulfate-polyacrylamide gel electrophoresis (SDS-PAGE) with an anticipated molecular weight 122 kDa.^20^

#### Mass Spectrometry

ESI–LC-MS/MS was performed at Virginia Commonwealth University Massey Cancer Center using a Q Exactive HFX (Thermo) coupled to an Easy nLC 1200 instrument (Thermo). The data were analyzed using the Sequest HT search algorithm using a custom *Lysinibacillus* database downloaded from SwissProt and processed with Proteome Discoverer 3.0.

## Results and Discussion

### *L. sphaericus* Precipitates Lithium Selectively over Sodium

To search for a microbial species capable of extracting minerals from evaporative salt brines, microbes were cultured from coal refuse collected from a mine in southwestern Virginia (United States), then screened for tolerance to various metal salts, and sent for 16s rDNA sequencing. When coincubated with lithium chloride in minimal media over a period of 48 h, *L. sphaericus* produced a solid, insoluble precipitate, while other microbes tested did not. While previous studies have shown *L. sphaericus* tolerance to and interaction with various transition metals,^20−22^ to the best of our knowledge there have not been any reports of *L. sphaericus* biomineralization with lithium.

## L. sphaericus

**Figure 1:**
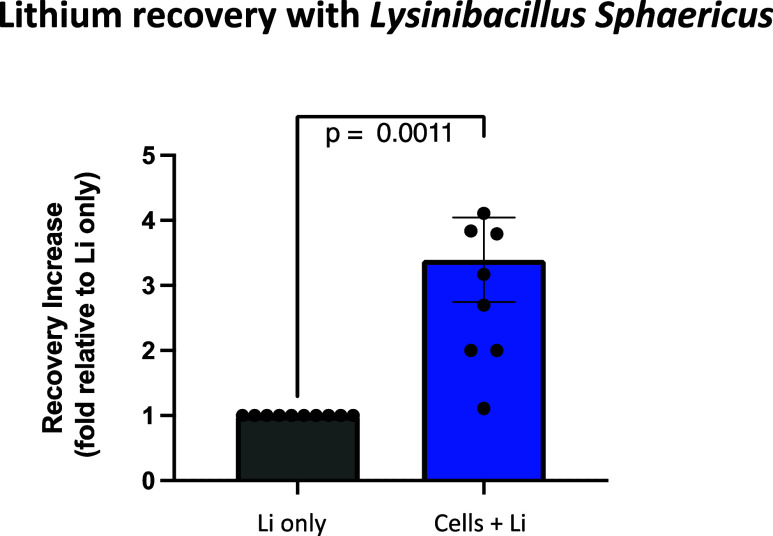
Lithium recovery following incubation with *L. sphaericus* cells as measured by HPBO fluorescent assay. “Li only” condition as compared to “Cells + Li” condition where “LiCl only” condition is normalized to 1. A two-tailed unpaired *t*-test between “Cells + Li” and “Li only” (df = 17) results in *p* = 0.0011.

Further experimentation with coincubation with lithium chloride shows significantly greater lithium precipitation with the microbe than without (Figure 1). No precipitation occurs when *L. sphaericus* is grown in the absence of a lithium salt. The precipitated lithium compound was isolated from solution via centrifugation, then lithium in the precipitate was quantified via fluorescent assay with 2-(2-hydroxyphenyl)-benzoxazole (HPBO).^23^ Obare and Murphy (2001) have shown that HPBO fluorescence is specific to complexation with lithium, and that sodium, potassium, calcium, and magnesium cations do not contribute to or interfere with the fluorescence signal. The specificity of HPBO fluorescence strongly suggests that the precipitate from *L. sphaericus* coincubation with lithium chloride is a lithium compound.

**Figure 2:**
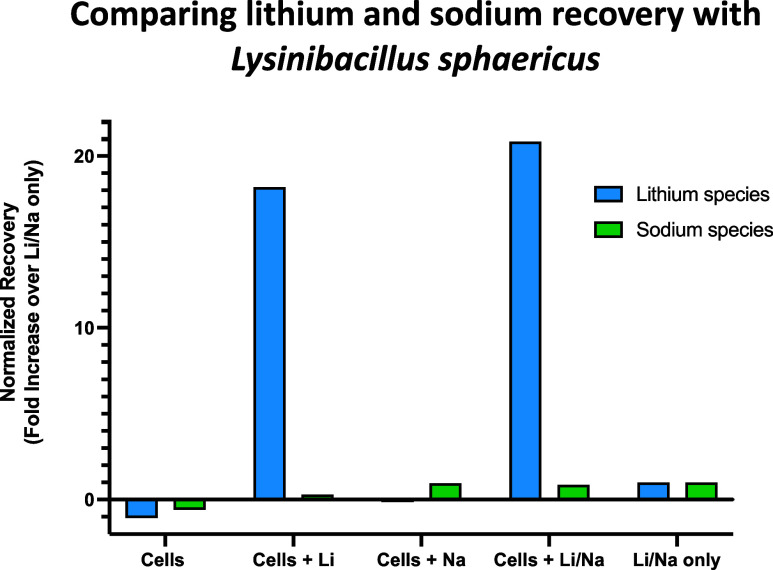
Lithium and sodium recovery following incubation with *L. sphaericus* cells as measured by HPBO fluorescent assay and CoroNa Green, respectively. *L. sphaericus* “Cells only” condition in comparison with “Cells + Li”, “Cells + Na”, “Cells + Li/Na”, and “Li/Na only” where “Li/Na only” condition is normalized to 1. A one-tailed paired *t*-test between HPBO and CoroNa Green (df = 7) results in *p* = 0.0862.

Since sodium is also found in high concentrations with lithium in salt brines,^3^ we tested *L. sphaericus* with sodium chloride under the same conditions. No precipitate resulted from coincubation of *L. sphaericus* with sodium chloride, suggesting that the microbe does not have any relevant interactions with sodium chloride. A sodium chloride and lithium chloride mixture was evaluated to determine whether the presence of sodium chloride interferes with *L. sphaericus* precipitation of lithium (Figure 2). Here, HPBO and CoroNa Green fluorescent assays were used to quantify lithium and sodium species, respectively. These results show that *L. sphaericus* precipitates lithium but not sodium from solution and that lithium precipitation is not hindered by the presence of sodium (Figure 2). The specificity of *L. sphaericus* mineralization with lithium is notable as lithium and sodium are difficult to separate chemically due to their similar charges and sizes.^24,25^

### Lithium Hydroxide Precipitated by *L. sphaericus* is in the Crystalline Nanoparticle Form

**Figure 3:**
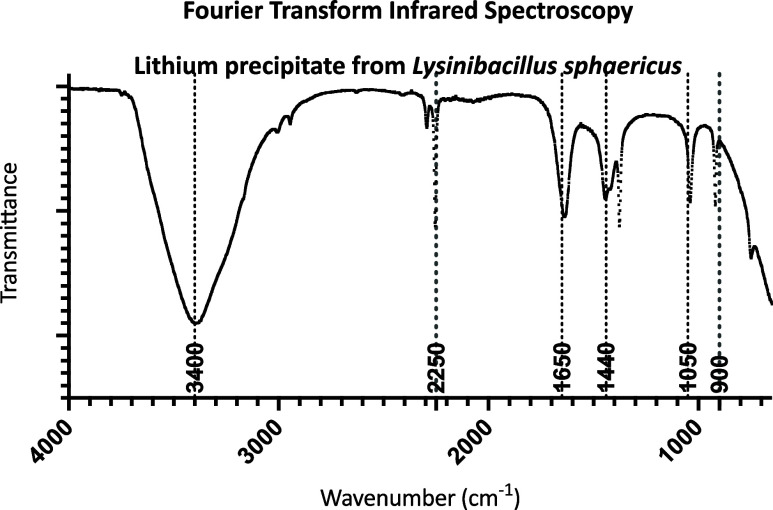
FTIR spectrum for lithium precipitate formed following coincubation with *L. spahericus*. Major peaks which correspond with lithium hydroxide reference spectra are labeled.

**Figure 4:**
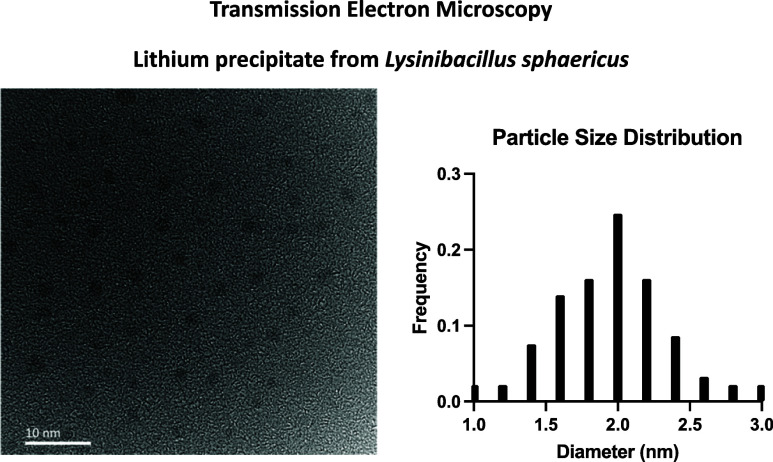
TEM for lithium precipitate formed following coincubation with *L. sphaericus*. Average particle size is 1.92 ± 0.42 nm. Frequency distribution of measured particle diameters (nm) organized by relative frequency (*n* = 93).

FTIR was used to determine the composition of the lithium precipitate. Figure 3 shows an FTIR spectrum with peaks at 3400, 2250, 1650, 1440, 1050, and 900 cm^–1^, which are all characteristic of lithium hydroxide and consistent with previous LiOH FTIR reports.^26,27^ The biological nature of this sample and absence of any postproduction purification steps contribute to the variation from known lithium hydroxide spectra. The size and morphology of precipitated lithium hydroxide were further evaluated with TEM. TEM shows spherical particles with an average diameter of 1.92 ± 0.42 nm (Figure 4), with an approximate polydispersity index of 0.2. The relatively narrow size distribution of particles suggests biomolecule capping, thus limiting any evolution of particle growth. Biomolecule capping of particles is often integral in biomineralization and is key for morphology and size control.^28−30^ To identify biomolecules potentially capping these particles, SDS-PAGE was used to separate a concentrated sample of *L. sphaericus*-synthesized lithium hydroxide particles and their associated biomolecules. This technique has been used previously to identify biomineralization enzyme smCSE.^31^ Three major bands are evident at approximately 80, 45, and 10 kDa (Figure S1). These bands were analyzed via ESI–LC-MS/MS, and the corresponding proteins are given in Table 1.

**Table 1:** Proteins Identified from SDS-PAGE of Concentrated Nanoparticles with ESI–LC-MS/MS

	protein	accession number (Swiss prot)	percent coverage	actual mass (kDa)
80 kDa band	S-layer homology domain containing protein	A0A7T2FPH4	36%	122
	catalase	A0A7T2FL42	28%	72
	aldehyde dehydrogenase family protein	A0A4U2YUK3	12%	31
	ATP synthase subunit alpha	A0A0A3H6SI	10%	55
	glyceraldehyde-3-phosphate dehydrogenase	A0A0A3IG21	12%	37
45 kDa band	chaperonin GroEL	A0A7T2CQ17	29%	57
	peptide ABC transporter substrate-binding protein	A0A7T2FPN4	17%	61
	oligopeptide ABC transporter substrate-binding protein	A0A7T2FPB5	18%	65
	catalase	A0A7T2FL42	11%	72
10 kDa band	flagellin	A0A0A3IGC1, A0A7T2FPL2	16%, 12%	29
	ZincABC transporter substrate-binding protein	A0A7T2C5D6	7%	34

#### S-Layer Protein Plays an Integral Role in Lithium Biomineralization

The S-layer protein, which makes up the outer surface layer of *L. sphaericus* cells, has an actual mass of 122 kDa, suggesting that the portion of S-layer protein shown at 80 kDa in the SDS-PAGE is a cleavage product. S-layer proteins have been identified and implicated in microbe–metal interactions previously, with purified S-layer proteins of *Bacillus sphaericus* JG-A12, mediating the formation of gold nanoclusters.^21^ The S-layer can function as a site for metal coordination and particle nucleation, which has been postulated as a protective mechanism for the cell to avoid metal toxicity.^32^ While many different bacteria have S-layers, not all S-layers exhibit biomineralization behavior.^32−34^

To evaluate the role of the S-layer protein in lithium biomineralization, we conducted biomineralization assays with *L. sphaericus* “ghosts”. A ghost refers to the membrane and S-layer of the cell without its contents, and such conditions have been thoroughly established previously by Pfeifer et al.^19^ Effectively, *L. sphaericus* ghosts have intact S-layers but none of the remaining proteins, as listed in Table 1.

**Figure 5:**
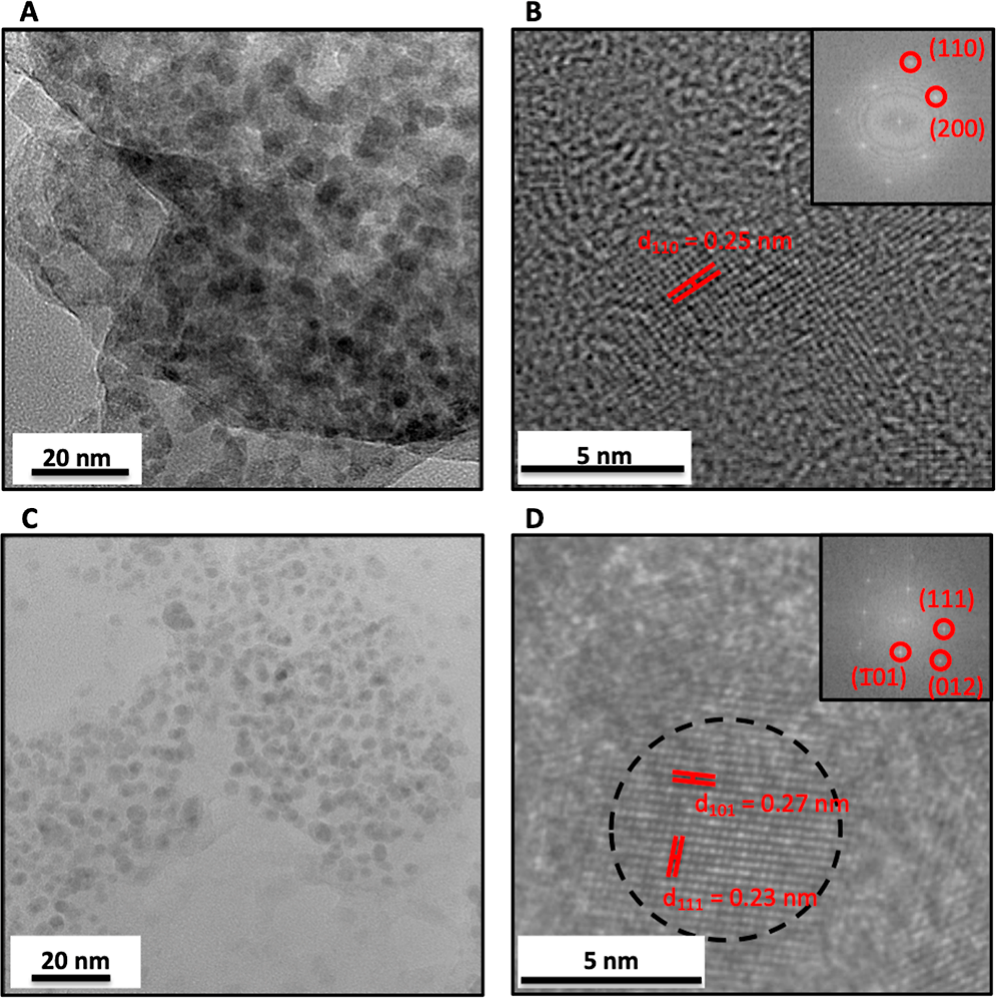
TEM for nanoparticles produced with *L. sphaericus*. (A) Particles were produced via incubation with live *L. sphaericus*. Scale bar is 20 nm. (B) Zoom view of a single particle produced via incubation with live *L. sphaericus*. Scale bar 5 nm. *D*-spacing along the (110) axis is 0.25 nm, consistent with lithium hydroxide. The inset shows Fourier transform with (110) and (200) spots indexed, indicating view down the (001) axis. (C) Particles produced with *L. sphaericus* ghosts. Scale bar is 20 nm. (D) Zoom view of a single particle produced via incubation with *L. sphaericus* ghosts. Schale is 5 nm. *D*-spacing (111) and (101) is 0.23 and 0.27 nm, respectively, consistent with lithium hydroxide. The inset shows Fourier transform with (111), (012), and (101) spots indexed, indicating a view down the (121) axis.

TEM imaging shows that nanoparticles are produced with both live *L. sphaericus* and *L. sphaericus* ghosts (Figure 5 a,c). Individual analysis and Fourier transforms of particles (Figure 5 b,d) reveal tetragonal crystalline lattices that match documented LiOH patterns (*P*4/*n m m*)^35^ with a slightly expanded lattice in the *c*-direction (*a* = *b* = 3.55 Å, *c* = 4.54 Å). The *d*-spacing for particles produced with live *L. sphaericus* is 0.25 nm, while *d*-spacing for particles produced with *L. sphaericus* ghosts was 0.27 and 0.23 nm along the (101) and (111) axis, respectively. The theoretical d101/d111 for crystalline lithium hydroxide is 1.22,^35−37^*L. sphaericus* ghost-produced particles exhibit d101/d111 of 1.17. The estimated *d*-spacings from the Fourier transform are 0.26 and 0.22 nm for (101) and (111), with a d101/d111 of 1.21, further supporting the identification of crystalline lithium hydroxide. Interestingly, the average particle diameter for *L. sphaericus* ghost-produced particles is 3.55 ± 0.8 nm (*n* = 88, Figure 5 c). As shown in Figure 4, the average particle diameter for live *L. sphaericus* produced particles is 1.92 ± 0.42 nm. Taken together, these findings suggest that the S-layer plays a critical role in the biomineralization of lithium chloride to lithium hydroxide; however, the involvement of other proteins in Table 1 may contribute to particle size control given the observed difference in nanoparticle size for intact cells versus ghosts.

#### Other Proteins may be Involved in *L. sphaericus* Biomineralization of Lithium

Of the remaining proteins listed in Table 1, catalase (accession no. A0A7T2FL42) was identified in both the 80 and 45 kDa bands, suggesting that it is closely associated with the nanoparticles. A previous study with environmental bacteria *Enterobacter cloacae* observes upregulation and overexpression of catalase as a cellular stress response to high metal concentrations (200 ppm).^38^ Catalase also requires a metal ion (usually iron) for its native activity,^39^ so it is possible there is some binding promiscuity with lithium. The possibility of catalase binding promiscuity with lithium is further supported as artificial metalloenzymes are an active area of research for biotechnological applications with copper (2+), cobalt (2+), and methyl-iridium (1+) replacing iron.^40,41^

Chaperonin GroEL (accession # A0A7T2CQ17) may also play a role in the lithium hydroxide nanoparticle production shown here as Chaperonin GroEL (various species) binds transition metals such as iron, nickel, cobalt, and palladium due to a high density of histidine residues central to the molecule.^42−44^ The cavity within Chaperonin GroEL has been calculated to be 175,000 cubic angstroms, with most substrates occupying 58,000 cubic angstroms.^45^ A simple calculation shows that 175,000 cubic angstroms is approximately 75 Å in spherical diameter, while 58,000 cubic angstroms is approximately 48 Å in spherical diameter. The 2 nm particles synthesized by *L. sphaericus* would therefore fit comfortably in the Chaperonin GroEL cavity. Chaperonin GroEL plays a significant role in cytosolic protein folding, and it is not cell membrane associated.^46^ Therefore, it would not be expected to accompany *L. sphaericus* ghosts through isolation.

Although many of these proteins have potential for biomineralization, it is likely that the biomineralization activity seen here results from an interplay between multiple proteins. One notable example is the biomineralization of magnetite in magnetotactic bacteria: a whole organelle with a multitude of proteins interact to guide magnetite formation.^47^ Aside from catalase and GroEL, there are documented interactions of aldehyde dehydrogenases,^48^ adenosine 5′-triphosphate (ATP) synthases,^49^ glyceraldehyde-3-phosphate dehydrogenases,^50^ and ABC transporter substrate-binding proteins^51−53^ with alkali earth metal magnesium and various transition metals including copper, cobalt, iron, nickel, and lead; each of these proteins is also identified in Table 1 as associated with biomineralized nanoparticles. Identifying the protein or proteins responsible for biomineralization with *L. sphaericus* may simplify future lithium extraction approaches.

## Environmental Relevance

The increase in global demand for lithium is expected to be economically disruptive and a great stressor to the global supply chain.^9^ Furthermore, current lithium extraction techniques such as mining and salt brines are resource intensive with negative environmental and community impacts.^6−8^ In this work, we explored lithium biomineralization via L. sphaericus for its potential use in lithium extraction and recovery as a microbial biomineralization approach. Compared to current lithium extraction techniques, biomineralization has the potential to be less time- and resource-intensive as well as more selective, limiting the downstream hydrometallurgical processing required.

The appearance of an insoluble precipitate following the incubation of *L. sphaericus* with aqueous lithium chloride highlights a useful microbial interaction that can be utilized for lithium recovery. Furthermore, we showed that lithium recovery is not inhibited by the inclusion of sodium ions in solution, which is promising for field applications. The proteins identified by ESI–LC–MS/MS each have potential to be key in the biomineralization process and provide solid avenues for further study regarding lithium biomineralization. Microscopic views of the particles produced with live *L. sphaericus* and *L. sphaericus* ghosts show that the S-layer plays an integral part in the biomineralization of lithium hydroxide nanoparticles but that other proteins may be involved in particle size control. Future work with *L. sphaericus* or individual proteins may be invaluable for lithium extraction and meeting the growing global demand for critical minerals.

## Data Availability

The original contributions presented in the study are included in the article/Supporting Information.

## Supporting Information Available

SDS-PAGE for nanoparticle-associated biomolecules used in ESI–LC-MS/MS (PDF)
