# Supplementary material for: Microbial Mineralization with Lysinibacillus sphaericus for Selective Lithium Nanoparticle Extraction
Source: Environ Sci Technol. 2024 Sep 12;58(38):16915–21. doi: 10.1021/acs.est.4c06540 (PMC11447963; doi:10.1021/acs.est.4c06540)
Supplement: Supplementary file 1 — es4c06540_si_001.pdf [file es4c06540_si_001.pdf]

Supplemental Information: Microbial mineralization with *Lysinibacillus sphaericus* for lithium nanoparticle extraction

Toriana N. Vigil,[a] Grayson C. Johnson,[a] Sarah G. Jacob,[b] Leah C. Spangler,[c] Bryan W. Berger[a]*

a. Department of Chemical Engineering, University of Virginia, Charlottesville, Virginia USA 22903

b. Department of Chemical and Biomolecular Engineering, University of Maryland, College Park, Maryland USA 20742

c. Department of Chemical and Life Science Engineering, Virginia Commonwealth University, Richmond, Virginia USA 23284

*Correspondence: bwb2k@virginia.edu

Contents	
Page S1	Figure S1. SDS-PAGE for concentrated nanoparticles produced via co-incubation with *Lysinibacillus sphaericus.* 80, 45, and 10 kDa bands were then analyzed via mass spectrometry.

SDS-PAGE for concentrated nanoparticles

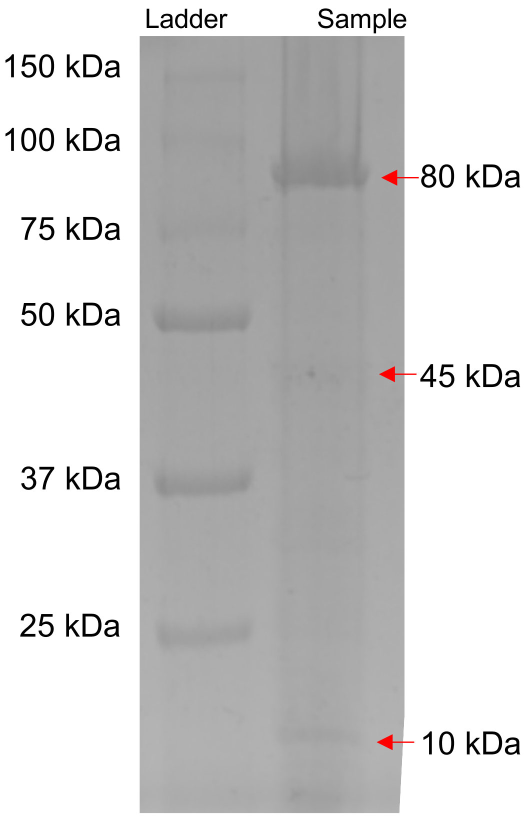

Supplemental Figure 1. SDS-PAGE for concentrated nanoparticles produced via co-incubation with *Lysinibacillus spahericus.* 80, 45, and 10 kDa bands were then analyzed via mass spectrometry.
